# Thoracic Duct Injury in a Patient Undergoing Axillary Lymphadenectomy: A Case Report

**DOI:** 10.1155/carm/9720159

**Published:** 2025-08-04

**Authors:** Michelle Albano Ferreira, Juliana Oliveira Costa, Juliana Lopes de Aguiar Araújo, Kleyton Santos de Medeiros, Larissa dos Santos Lourenço Ferreira, Ubiratan Wagner de Sousa, Macerly Layse de Menezes Dantas, Diana Taissa Sampaio Marinho Navarro

**Affiliations:** ^1^Department of Mastology, League Against Cancer, Natal, Rio Grande do Norte, Brazil; ^2^Department of Medicine, Potiguar University, Natal, Rio Grande do Norte, Brazil; ^3^Institute of Education, Research and Innovation, League Against Cancer, Natal, Rio Grande do Norte, Brazil

**Keywords:** breast cancer, chyle, thoracic duct

## Abstract

A 56-year-old female patient, with no significant comorbidities, presented with abnormal breast exam findings. Imaging revealed a 5.4-cm irregular nodule in the left breast, diagnosed as invasive breast carcinoma (NST, Grade 2). Neoadjuvant chemotherapy was initiated, leading to a reduction in lesion size. Surgical intervention included quadrantectomy, sentinel lymph node biopsy, and axillary lymphadenectomy, which revealed residual carcinoma and positive lymph nodes. Postoperatively, chylous drainage through a Portovac drain was observed, prompting reoperation, during which the injured lymphatic duct was identified. Conservative management with medium-chain triglycerides resulted in a progressive reduction of drainage. The patient was discharged on the 13th postoperative day, subsequently underwent adjuvant radiotherapy, and is currently receiving regular outpatient follow-up.

## 1. Introduction

The thoracic duct is responsible for transporting lymph from the intestines to the bloodstream. It originates in the cisterna chyli, the convergence of the intra-abdominal lymphatics, and drains into the junction of the left subclavian and jugular veins. Surgical injuries to this structure have been reported following thoracic surgical procedures, particularly those involving the left upper thoracic duct, which may inadvertently interrupt the thoracic duct or lacerate its lymphatic tributaries [1].

Anatomical variations may occur at the termination of the thoracic duct, rendering it susceptible to injury during axillary dissection [2]. Injury to the branching tributaries of the thoracic duct may necessitate prolonged management, resulting in significant costs for both the patient and medical staff. Chyle fistula can cause extreme morbidity because of the loss of fluids, electrolytes, and other nutrients [3]. Besides that, thoracic duct laceration is a rare but potentially life-threatening complication. The management of such an injury is uncertain in respect of the relative merits of conservative and surgical treatment [4].

Considering the rarity of the case with an incidence of less than 0.5% [3] and the limited number of reports on this topic in the literature, this article aims to present a clinical case of a patient with an injury to the thoracic duct or its tributary lymphatic vessels following axillary lymphadenectomy.

## 2. Case Presentation

A 56-year-old female patient, an ex-smoker who has been abstinent for 25 years, was admitted to the mastology department due to abnormal exam results. She had no significant comorbidities or relevant family history, except for a paternal history of prostate cancer. On physical examination, an area of thickening with a hardened, nodular appearance was observed in the lateral quadrants of the left breast, measuring approximately 8 cm. Regarding imaging studies, breast ultrasound (USG) revealed a 5.4-cm nodule in the LB, located between the 3 and 4 o'clock positions, 7 cm from the nipple, with an irregular appearance (BI-RADS 5). Mammography showed dense breast tissue with benign microcalcifications (BI-RADS 2).

She underwent a core needle biopsy, the histopathological results of which were consistent with invasive breast carcinoma of no special type (NST), histological Grade 2. Immunohistochemistry revealed a luminal B subtype (ER 90%; PR 40%; HER2 0; Ki67 30%).

Given the disproportion between tumor size and breast volume, it was decided to initiate treatment with neoadjuvant chemotherapy. The patient underwent four cycles of adriamycin and cyclophosphamide, followed by twelve cycles of paclitaxel. Staging exams revealed no evidence of metastatic disease.

At the completion of neoadjuvant therapy, a reduction in the lesion size was noted. Physical examination revealed an area of poorly defined thickening, and ultrasound demonstrated a residual lesion measuring 2.4 cm. Mammography revealed an area of distortion associated with pleomorphic microcalcifications in the posterior third of the left lower quadrants of the left breast.

Consequently, a surgical approach was indicated, involving breast quadrantectomy and biopsy of the left sentinel lymph node. Histopathological examination of the specimen confirmed histological Grade 2 invasive breast carcinoma of no special type (NST-CIN), associated with high-grade ductal carcinoma in situ (DCIS G3). The microscopic dimension of the residual invasive neoplasia was 2 cm, with focal involvement of the lower and deep margins. In the sentinel lymph nodes, two of the four biopsied were positive, without extracapsular extension (Ep: ypT1cN1mi). As a result, surgical margin enlargement and axillary lymphadenectomy were performed.

**Figure 1:**
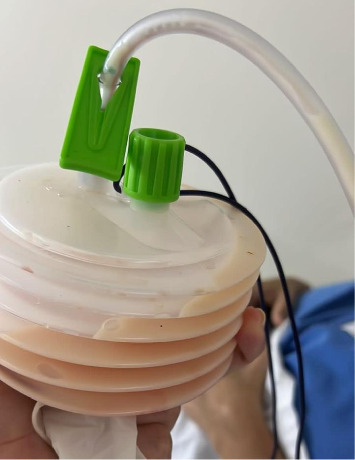
Output and chylous coloration of the drainage secretion in the immediate postoperative period.

During axillary lymphadenectomy, the anatomical boundaries were carefully observed, preserving critical structures such as Bell's nerve, the brachial plexus, and the axillary vein. Subsequently, a 6.4-mm Portovac drain was inserted and secured. The procedure proceeded without complications. On the first postoperative day, the patient exhibited drainage of chylous secretion through the Portovac drain, with a flow rate of 370 mL (Figure 1). A thoracic surgery consultation was requested due to suspected lymphatic duct injury, and the patient was advised to withhold oral intake, initiate supplementation with a medium-chain triglyceride-rich diet, and schedule a follow-up to attempt identification of the lymphatic duct.

**Figure 2:**
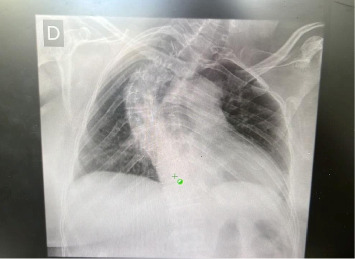
Chest X-ray demonstrated severe scoliosis and alterations in the rib cage.

An analysis of the drain secretion was requested, and the triglyceride level was found to be 750 mg/dL. Additionally, a chest X-ray revealed significant scoliosis and alterations in the rib cage (Figure 2).

On the second postoperative day, the drain output remained elevated at 1050 mL/24 h. The following day, despite dietary adjustments, the drain output was 820 mL/24 h. A reoperation was subsequently scheduled, following the surgical preparation guidelines recommended by the thoracic surgery team: administer 200 mL of fatty food, such as olive oil or cream, 1 hour before the procedure to stimulate secretion production, and facilitate the identification of the injured duct during reoperation.

On the fourth postoperative day, axillary reoperation was performed. In addition to the milk cream, a small amount of water with methylene blue was administered. A whitish chylous secretion was identified in the region of the axillary apex near Level 3, where a Greek Bar suture was placed using Vicryl 3.0. A new Portovac drain was then inserted.

Following the reoperation, it was noted that, although the output had decreased, chylous secretion continued to drain at a rate of 800 mL/24 h. Conservative management was therefore maintained, consisting of a zero diet, dietary supplementation with medium-chain triglycerides, and the application of cold compresses to the site. On the second POD following the reoperation, the drain output decreased to 335 mL/24 h. From that point onward, the drain output progressively diminished, and the secretion changed from chylous/hematic to serous.

**Figure 3:**
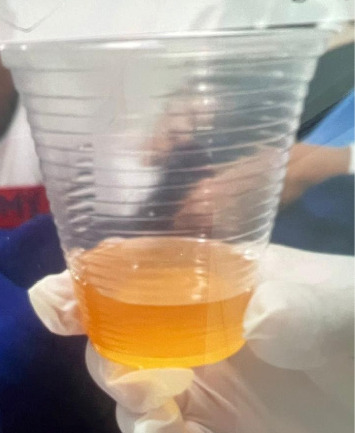
Color of the serous secretion on the day of drain removal (15th postoperative day).

The patient was discharged on the 13th POD after axillary lymphadenectomy and the 8th POD after axillary reoperation. The surgical wounds had well-coapted margins, and a Portovac drain was in place with a serous output of 30 mL/24 h (Figure 3). The patient was advised to follow a low-glycemic diet, and the nutrition team provided supplementation based on medium-chain triglycerides.

After discharge, the patient maintained the prescribed diet for 1 month. On the 15th postoperative day, the Portovac drain was removed. She was subsequently referred to as adjuvant radiotherapy, with 15 fractions of 267 cGy recommended. The patient is currently undergoing outpatient follow-up, with six-monthly visits while on tamoxifen, and reports no complaints.

## 3. Discussion

Injuries to the thoracic duct or its tributary lymphatic vessels primarily result from surgery or blunt trauma to the chest. Such injuries associated with axillary surgeries are rare [5]. The literature describes both conservative and surgical approaches for the management of these injuries [6].

The incidence of thoracic duct or tributary lymphatic duct injuries in axillary surgeries is less than 0.7% [1, 2]. These injuries occur most frequently on the left side of the chest and are closely associated with anatomical variations of these ducts [7].

The average age of patients with this condition is 53 years, and the average time between the initial axillary surgery and resolution of the condition is 17 days [8]. Typically, no adverse effects or sequelae are observed during the follow-up of oncological treatment [9].

The diagnosis is generally clinical, based on the observation of a milky, high-volume drain output. A biochemical evaluation of the drain contents, typically through lipid profiling, is performed to confirm the diagnosis. Additionally, computed tomography or lymphoscintigraphy may be employed to locate the fistula [6].

The literature indicates that in approximately 25%–50% of cases, spontaneous closure of the fistula occurs with conservative measures, following the initiation of parenteral nutrition or an enteral diet enriched with medium-chain triglycerides. In refractory cases, where there is no clinical response, a surgical approach is indicated, which may involve ligation of the thoracic duct or percutaneous embolization of the duct. Surgical treatment typically consists of video thoracoscopy or right thoracotomy to identify the lymphatic duct, followed by its ligation [9]. An alternative approach would be to ligate the tributary lymphatics in the axillary region, if identifiable, as was performed in the present case.

Conservative treatment is typically initiated upon diagnosis and consists of parenteral nutrition, or an enteral diet enriched with medium-chain triglycerides, in conjunction with the use of compresses and bed rest [9].

In most cases involving patients with high drainage output, the treatment approach includes complete intestinal rest through the administration of parenteral nutrition, aggressive correction of fluid and electrolyte imbalances, and, if necessary, ligation of the thoracic duct or percutaneous embolization of the duct [2].

Initial treatment with low-fat oral diets offers no benefit for high-output postoperative chyle leaks. However, an oral diet enriched with medium-chain triglycerides may serve as transitional nutrition for patients who have experienced a reduction in chyle drainage (< 500 mL/day) following initial management with parenteral nutrition and bowel rest [8].

The literature also describes the use of somatostatin or octreotide, a synthetic analog of somatostatin, as adjunctive treatments [10]. These medications reduce chyle production, lymph flow, and intestinal fat absorption [6]. However, these medications were not available in our service.

In the case described, an attempt was made to surgically ligate the tributary thoracic duct, but this procedure resulted in only partial resolution. Subsequently, maintaining an oral diet enriched with medium-chain triglycerides proved effective in achieving complete closure of the fistula and resolving the case within 13 days.

## 4. Conclusion

This is a case report of a patient with injury to the thoracic duct or its tributary lymphatic vessels following axillary lymphadenectomy. This case is significant as it highlights the occurrence of this rare surgical complication and contributes to the existing literature on the subject. Most axillary lymphatic leaks are managed conservatively; however, surgical intervention is required in cases of significant lymphatic leakage.

Early recognition and individualized management of the patient are crucial for achieving optimal follow-up. It is essential for specialists in the field of mastology to be aware of this complication and its most effective management strategies. A multidisciplinary approach, involving expertise from nutrition, thoracic surgery, radiology, and the nursing team, is required for the best outcomes.

## Data Availability

This is a case report in which the data are available in this material.
